# ARF induction in response to DNA strand breaks is regulated by PARP1

**DOI:** 10.1093/nar/gkt1185

**Published:** 2013-11-29

**Authors:** Giulia Orlando, Svetlana V. Khoronenkova, Irina I. Dianova, Jason L. Parsons, Grigory L. Dianov

**Affiliations:** Department of Oncology, Gray Institute for Radiation Oncology and Biology, University of Oxford, Roosevelt Drive, Oxford OX3 7DQ, UK

## Abstract

The ARF tumour suppressor protein, the gene of which is frequently mutated in many human cancers, plays an important role in the cellular stress response by orchestrating up-regulation of p53 protein and consequently promoting cell-cycle delay. Although p53 protein function has been clearly linked to the cellular DNA damage response, the role of ARF protein in this process is unclear. Here, we report that *arf* gene transcription is induced by DNA strand breaks (SBs) and that ARF protein accumulates in response to persistent DNA damage. We discovered that poly(ADP-ribose) synthesis catalysed by PARP1 at the sites of unrepaired SBs activates ARF transcription through a protein signalling cascade, including the NAD^+^-dependent deacetylase SIRT1 and the transcription factor E2F1. Our data suggest that poly(ADP-ribose) synthesis at the sites of SBs initiates DNA damage signal transduction by reducing the cellular concentration of NAD^+^, thus down-regulating SIRT1 activity and consequently activating E2F1-dependent ARF transcription. Our findings suggest a vital role for ARF in DNA damage signalling, and furthermore explain the critical requirement for ARF inactivation in cancer cells, which are frequently deficient in DNA repair and accumulate DNA damage.

## INTRODUCTION

The tumour suppressor protein ARF is a key player involved in regulation of p53 protein levels in mammalian cells, and the *arf* gene is frequently inactivated in many human cancers (1,2). ARF is also implicated in cellular senescence and has been reported to accumulate during aging (3). The major function of ARF protein is the transmission of stress-induced signals to proteins executing the stress response, and the initiation of programmed death (apoptosis) of genetically unstable cells. The E3 ubiquitin ligase Mdm2, which controls p53 levels and thus regulates many cellular stress responses, is the major target of ARF protein (4). Another important target is the E3 ubiquitin ligase ARF-BP1/Mule which is also involved in regulation of p53 (5), DNA repair (6–8) and apoptosis (9). However, it is widely accepted that ARF plays a major role in the cellular stress response by inhibiting Mdm2, which promotes p53 ubiquitylation and subsequent proteasomal degradation. Therefore, inhibition of Mdm2 by ARF leads to p53 accumulation, which results in either a cell-cycle delay required for DNA repair, or induction of apoptosis (10). Several studies have shown that ARF is up-regulated in response to oncogenic stress (11–13); however, it is not clear whether ARF is induced by DNA damage, one of the most well-documented activators of the p53-dependent stress response (1,2). An early report from the Alt laboratory suggested that ARF is inducible in response to chronic genotoxic stress; however, the induction mechanism was not investigated in detail (14).

As a well-known molecular sensor of SBs, PARP1 protein was originally thought to be a DNA damage detecting molecule that initiates chromatin remodelling and to also assist in assembly of DNA repair complexes at the sites of DNA damage (15). Earlier studies also suggested that PARP1 may be a DNA damage signalling molecule that is required for initiation of the cellular DNA damage response and consequent activation of p53, although the mechanism involved was not investigated (16–18). PARP1 is a poly(ADP-ribose) polymerase with a high binding affinity to SBs. When bound to SB as a dimer, PARP1 uses NAD^+^ molecules as building blocks to synthesize long polymers of poly(ADP-ribose) (PAR), primarily at the partner molecule within the dimer. When negatively charged PAR polymers are sufficiently long, this forces PARP1 dimer dissociation from the DNA and allows access of the SB to DNA repair enzymes. A new, unmodified PARP1 molecule may bind to the same SB again if repair is not accomplished after the first round and, as a result of this, multiple cycles of PARP1-binding dissociation on unrepaired SBs may reduce cellular NAD^+^ concentrations (19,20). Thus, quantitation of intracellular NAD^+^ can be used to monitor an imbalance of DNA single strand break (SB) repair in base excision repair (BER) deficient cells in real time (21). It was previously proposed by several authors that NAD^+^ depletion may deactivate NAD^+^-dependent cellular stress response proteins. In particular, a connection between PARP1 and SIRT1 protein, a NAD^+^-dependent deacetylase, was proposed (22). Moreover, it was independently demonstrated that ARF expression is regulated by the transcription factor E2F1, whose activity is in turn controlled by SIRT1 (23–27). Surprisingly, however, the link between PARP1, SIRT1, E2F1 and ARF induction by SBs has not been established. In this study, we identified PARP1, SIRT1 and E2F1 as components of the DNA damage transmission pathway induced by unrepaired SBs. Our data suggest that PAR synthesis by PARP1 at the sites of unrepaired SBs initiates DNA damage signal transduction by depleting the NAD^+^ pool, thus reducing SIRT1 activity and consequently activating E2F1-dependent *arf* transcription.

## MATERIALS AND METHODS

### Western blots

Western blots were performed by standard procedure as recommended by the vendor (Novex, San Diego, USA). Blots were visualized and quantified using the Odyssey image analysis system (Li-Cor Biosciences, Cambridge, UK). Sources of the antibodies used are summarized in Supplementary Table S1.

### Plasmids

For over-expression experiments, pCMVHA E2F1 (Addgene plasmid number: 24225) and pCMV3Tag3a XRCC1 expression vectors were used. To generate siRNA resistant mutant a 2-nt mutation was introduced into XRCC1 expression vector using the QuikChange® Site-Directed Mutagenesis Kit from Agilent Technologies. Mutants were verified by sequencing.

### Whole-cell extracts

Whole-cell extracts were prepared by Tanaka’s method (28). Briefly, cells were re-suspended in one packed cell volume of buffer containing 10 mM Tris–HCl (pH 7.8), 200 mM KCl, 1 mg/ml of each protease inhibitor (pepstatin, aprotinin, chymostatin and leupeptin), 1 mM PMSF and 1 mM NEM. Two packed cell volumes of buffer containing 10 mM Tris–HCl (pH 7.8), 600 mM KCl, 40% glycerol, 0.1 mM EDTA and 0.2% Nonidet P-40 was then added and mixed thoroughly before rocking the cell suspension for 2 h at 4°C. The cell lysate was then centrifuged at 40 000 rpm at 4°C for 20 min and the supernatant was collected, aliquoted and stored at −80°C.

### Alkaline single-cell gel electrophoresis (Comet) assay

The comet assay was performed as recently described (7). Briefly, cells were trypsinised, treated or mock-treated in suspension with hydrogen peroxide for 5 min or irradiated (10 Gy) on ice. Cells were embedded on a microscope slide in agarose (Bio-Rad, Hemel Hempstead, UK) and the slides were incubated for various times at 37°C in a humidified chamber to allow for DNA repair. The slides were subsequently placed in lysis buffer containing 2.5 M NaCl, 100 mM EDTA, 10 mM Tris–HCl pH 10.5, 1% (v/v) DMSO and 1% (v/v) Triton X-100 for 1 h at 4°C. The slides were then incubated in the dark for 30 min in cold electrophoresis buffer [300 mM NaOH, 1 mM EDTA, 1% (v/v) DMSO, pH 13] to allow the DNA to unwind prior to electrophoresis at 25 V for 25 min. After neutralisation with 0.5 M Tris–HCl (pH 8.0), the slides were stained with SYBR Gold (Invitrogen, Paisley, UK) and analysed using the Komet 5.5 image analysis software (Andor Technology, Belfast, Northern Ireland).

### FACS analysis

Harvested cells were fixed in ice-cold 70% ethanol for at least 30 min. After removal of the fixative solution by centrifugation, cells were treated for 30 min at 37°C with 20–100 μg/ml RNAse in PBS and then stained in PBS containing 10 μg/ml propidium iodide (Sigma). Samples were run on a Becton-Dickinson FACScan (BD Biosciences, Oxford, UK) and the obtained data were analysed using Modfit LT software (Verity Software House).

### Luciferase assay

HeLa cells (2 × 10^4^ cells) were seeded per well of the 24-well plate for 24 h. For gene silencing, cells were treated with Lipofectamine transfection reagent in the absence (Contr) or presence of 0.2 pmol XRCC1 siRNA (XRCC1 RNAi) for 48 h. The cells were further transfected with 200 ng p14ARF luciferase reporter plasmid (*p14ARF* −388/+22) kindly provided by Dr K. Yoshida, 200 ng pCMVHA-E2F1 or control empty vector (pCMVHA), and 10 ng pRL-TK Renilla luciferase reporter plasmid (Promega) as an internal control using Lipofectamine transfection reagent for 24 h. Cells were then lysed in 100 µl passive lysis buffer and the extracts (10 µl) were assayed for luciferase activity using a Dual Luciferase Reporter Assay kit (Promega). The measurements were performed in triplicate. Light intensity was measured using a MicroLumat *Plus* LB 96B luminometer (Berthold Technologies, Germany). The obtained firefly luciferase activity values were normalized to the corresponding activity values of *Renilla* luciferase to control for the transfection efficiency. The activity of E2F1-regulated *p14ARF* −388/+22 luciferase reporter was presented as fold activation relative to luciferase activity in the absence of E2F1 expressing plasmid (pCMVHA control empty vector).

### Real-time PCR

Total RNA was purified using the RNeasy kit (Qiagen) and cDNA was prepared using the SuperScript RT–PCR system (Invitrogen). Quantitative rtPCR was performed using Absolute Blue QPCR SYBR low ROX Mix (Thermo Scientific) according to the manufacturer’s protocol and reactions were carried out in triplicate for each target transcript using a 7500 Fast Real-Time PCR System (Applied Biosystems). The comparative C_T_ method was applied for quantification of gene expression, values were normalised against GAPDH as a control and results expressed as fold change in mRNA levels. Real-time PCR was performed using the following primers: p14ARF forward (5′CTACTGAGGAGCCAGCGTCTA3′), p14ARF reverse (5′CTGCCCATCATCATGACCT3′); GAPDH forward (5′AGCCACATCGCTCAGACAC3′) GAPDH reverse (5′GCCCAATACGACCAAATCC3′).

### NAD^+^ level

Cells were trypsinized and 2 × 10^5^ cells were used for analysing NAD^+^ levels, according to the manufacturer’s protocol (Abcam). The assay was performed in triplicate. To quantify the results, absorbance at 450 nm was measured using a Polastar Omega plate reader (BMG Labtech).

### Statistical analysis

Statistical data are presented as a mean ± SD of at least three independent biological experiments and *P*-values were calculated by Student’s *t*-test (*P < 0.05, **P < 0.01 and ***P < 0.001).

### Gene silencing

For gene silencing, cells were transfected with the corresponding siRNA using Lipofectamine RNAiMAX reagent (Invitrogen) according to the manufacturer’s protocol and harvested after 72 h. The siRNA sequences used are shown in Supplementary Table S2. Where indicated, cells were subjected to ionizing radiation using GSR-D1 137Cs γ-irradiator (RPS Services Limited) at a dose rate of 1.8 Gy/min (10 Gy dose) or treated with 150 µM hydrogen peroxide for 15 min at 37°C.

## RESULTS

### Persistent DNA SBs induce ARF accumulation

**Figure 1.**
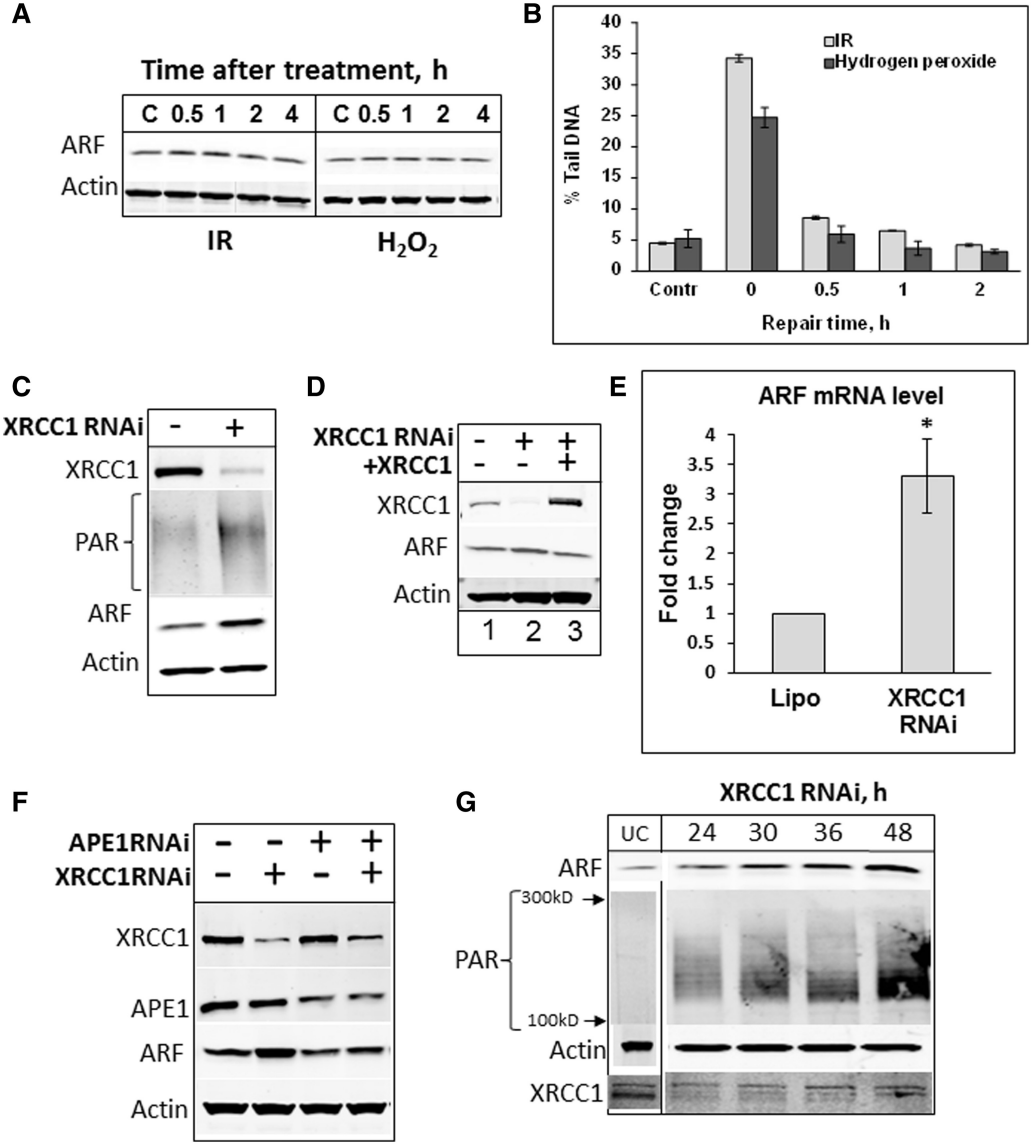
Transcription of ARF is activated in response to persistent DNA SBs. **(A)** Neither irradiation nor hydrogen peroxide treatment significantly increase ARF protein levels. HeLa cells were either untreated (Control, **C**) or treated with 150 µM hydrogen peroxide (H_2_O_2_) for 15 min or with 10 Gy ionizing radiation (IR) and allowed to recover for the time indicated. Whole-cell extracts were prepared and analysed by SDS–PAGE and immunoblotting with the antibodies indicated. **(B)** Alternatively following treatment, DNA repair was assessed by the alkaline single cell gel electrophoresis (Comet) assay. **(C)** Generation of persistent SBs by an XRCC1 knockdown induces ARF protein. HeLa cells were treated with Lipofectamine transfection reagent in the absence and presence of XRCC1 siRNA (200 pmol) for 72 h. Whole-cell extracts were prepared and analysed by SDS–PAGE and immunoblotting with the indicated antibodies. (**D**) Expression of siRNA resistant XRCC1 protein prevents ARF induction generated by a knockdown of XRCC1. HeLa cells were treated with Lipofectamine transfection reagent in the absence and presence of XRCC1 siRNA (200 pmol) for 24 h followed by a further 24 h treatment with Lipofectamine transfection reagent in the presence of mammalian expression plasmids expressing siRNA-resistant XRCC1 protein (600 ng). Whole-cell extracts were prepared and analysed by SDS–PAGE and immunoblotting. (**E**) Increased transcription of *arf* gene following XRCC1 knockdown. HeLa cells were treated with Lipofectamine transfection reagent in the absence and presence of XRCC1 siRNA (200 pmol) for 72 h. mRNA was prepared and expression of *arf* gene was analysed by real-time PCR. Statistical data are presented as a mean ± SD of three independent biological experiments and *P*-values were calculated by Student’s *t*-test (*P < 0.05). (**F**) APE1 knockdown reduces the induction of ARF by persistent SBs. HeLa cells were treated with Lipofectamine transfection reagent in the absence and presence of XRCC1, APE1 or both siRNA, (200 pmol each) for 72 h. Whole-cell extracts were prepared and analysed by SDS–PAGE and immunoblotting with the indicated antibodies. (**G**) Kinetics of PAR and ARF induction following XRCC1 knockdown. HeLa cells were treated with Lipofectamine transfection reagent in the presence of XRCC1 siRNA (200 pmol). Cells were harvested at the indicated times, whole-cell extracts were prepared and analysed by SDS–PAGE and immunoblotting with the indicated antibodies. ‘UC’ stands for untreated cells control.

In normal cells, which have efficient DNA repair mechanisms that maintain low levels of endogenous DNA damage, ARF protein is hardly detectable (29). We used HeLa cells as a model system for studying the mechanisms of regulation of ARF protein levels, since they express moderate levels of ARF that can be modulated up or down. Furthermore, up-regulation of ARF can be tolerated in these cells as it does not trigger cell-cycle delay and significant cell death, since p53 accumulation induced by ARF is kept under control by the presence of the human papilloma virus E6 and E6AP proteins that promote p53 degradation. In agreement with previously published data (30), we detected only a minor increase (15–20%) in the protein levels of ARF in HeLa cells in response to ionizing radiation or oxidative DNA damage (Figure 1A). However, in both cases we clearly detected formation of a significant amount of SBs that were repaired within the first hour post-treatment, as measured by the Comet assay (Figure 1B). We thus concluded that DNA damage itself, if it is promptly repaired, modulates only minor fluctuations in ARF protein levels and does not lead to its accumulation. We next hypothesized that the previously reported accumulation of ARF during cell senescence and aging (3,31) is triggered by persistent unrepaired DNA lesions, which are known to accumulate over multiple rounds of cell proliferation (32). To promote the accumulation of persistent DNA damage in HeLa cells we disabled the BER pathway by an siRNA knockdown of XRCC1, a scaffold protein controlling assembly of DNA repair complexes (33). BER is responsible for the repair of damaged DNA bases and single SBs that are generated in their thousands in every cell due to chemical instability of the DNA molecule, but are also induced by intracellular mutagens (34). Cells deficient in XRCC1 have previously been demonstrated to contain an increased level of SBs, mainly DNA single SBs, without additional DNA damage treatment (33). The formation of SBs in HeLa cells following an XRCC1 knockdown was confirmed by the accumulation of PAR, the synthesis of which is known to be activated by SBs (Figure 1C). In support of our hypothesis, persistent SBs caused by an XRCC1 knockdown in HeLa cells consistently resulted in a 3.4 ± 0.8-fold increase in ARF protein levels (Figure 1C and Supplementary Figure S1). To demonstrate that ARF accumulation is owing to a deficiency in XRCC1 rather than caused by an unspecific effect of the siRNA, we transfected XRCC1 knockdown cells with a plasmid expressing siRNA resistant *xrcc1* gene and observed decreased ARF induction (Figure 1D). We did not observe any significant changes in ARF protein stability following an XRCC1 knockdown (Supplementary Figure S2), suggesting that the up-regulation of ARF in response to persistent SBs mainly occurred at the transcriptional level. Indeed, quantitative PCR analyses detected a 3- to 4-fold increase in *arf* mRNA levels following an XRCC1 knockdown (Figure 1E). To further verify that ARF protein induction is due to accumulation of SBs we partially knocked down AP-endonuclease (full knockdown is lethal for cells), the enzyme that generates SBs during BER. As expected, we observed a reduced induction of ARF under these conditions in comparison to an XRCC1 knockdown (Figure 1F). Importantly, although an XRCC1 knockdown was already achieved after 24 h, the amount of ARF protein linearly increased over time in synchrony with PAR accumulation, reflecting time-dependent accumulation of endogenous SBs (Figure 1G). We thus concluded that unrepaired SBs accumulating following an XRCC1 knockdown stimulates *arf* transcription.

### PARP1, SIRT1 and E2F1 are required for ARF induction by SBs

**Figure 2.**
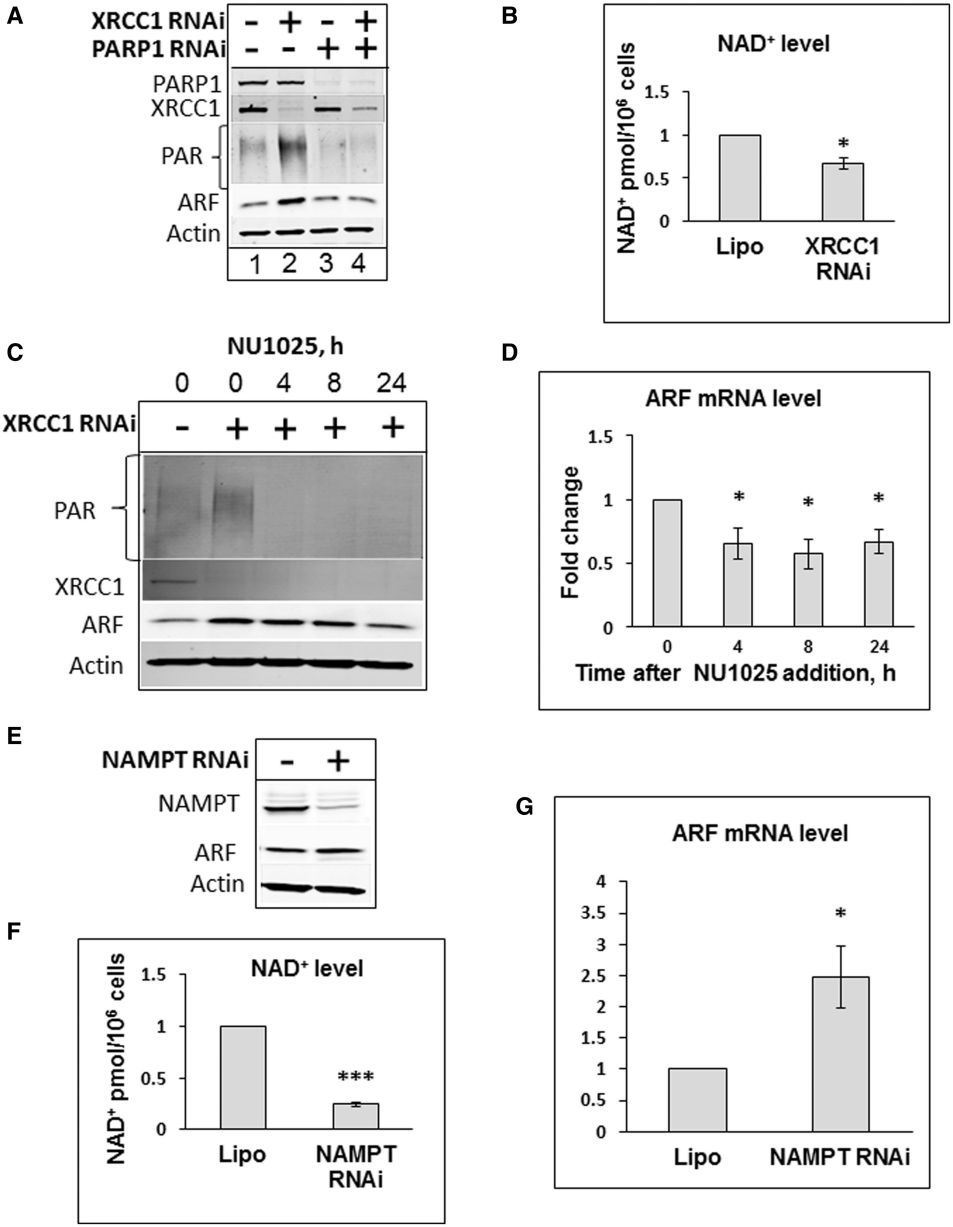
PARP1 activates *arf* gene transcription by modulating cellular NAD^+^ levels. (**A**) PARP1 is required for XRCC1 knockdown-induced ARF protein accumulation. HeLa cells were treated with Lipofectamine transfection reagent in the absence and presence of XRCC1 or PARP1 siRNA (200 pmol), or a combination of both for 72 h. (**B**) Reduction of NAD^+^ levels after XRCC1 knockdown. XRCC1 was knocked down for 48 h and NAD^+^ levels were analysed using a colorimetric assay. (**C** and **D**) Inhibition of PAR synthesis reduces ARF levels and *arf* transcription. HeLa cells were treated with Lipofectamine transfection reagent (10 µl) in the absence and presence of XRCC1 siRNA (200 pmol) for 72 h. Cells were further treated with 200 µM of PARP1 inhibitor NU1025 for the indicated time. Whole-cell extracts were prepared and analysed by SDS–PAGE and immunoblotting with the indicated antibodies (C). mRNA was prepared and expression of *arf* gene was analysed by real-time PCR (D). (**E–G)** NAMPT knockdown reduces cellular NAD+ levels and promotes *arf* transcription. HeLa cells were treated with Lipofectamine transfection reagent in the absence and presence of NAMPT siRNA (200 pmol). Whole-cell extracts were prepared and analysed by SDS–PAGE and immunoblotting with the indicated antibodies. Cellular levels of NAD^+^ were analysed using a colorimetric assay and *arf* transcription levels were analysed by real-time PCR. Statistical data are presented as a mean ± SD of three independent biological experiments and *P*-values were calculated by Student’s *t*-test (*P < 0.05; ***P < 0.001).

It is known that PARP1 is the major sensor of SBs, that upon binding, initiates synthesis of PAR (15,35). We next hypothesized that PAR synthesis by PARP1, in response to unrepaired SSBs, is required for activation of *arf* transcription and accumulation of ARF protein. To test this hypothesis, we performed a siRNA knockdown of PARP1 in combination with XRCC1, and observed that the elevation of ARF levels induced by an XRCC1 knockdown (Figure 2A, lane 2) was effectively blocked by a simultaneous PARP1 knockdown (Figure 2A, lane 4). We thus concluded that PARP1, and possibly consumption of NAD^+^ stimulated by PARP1 activity following SB binding, is required for ARF induction. To further test this, we first demonstrated that an XRCC1 knockdown in our experimental system does indeed result in a reduction of the cellular concentration of NAD^+^ (Figure 2B). We also showed that inhibition of PARP1 activity by the PAR synthesis inhibitor NU1025, although it did not have an immediate effect on ARF protein level (we observed only a gradual ARF protein decline; Figure 2C), resulted in an instant decrease in *arf* gene transcription (Figure 2D). We thus concluded that activation of *arf* gene transcription is as a result of reduced cellular NAD^+^ concentration. To directly challenge this model, we knocked down the NAD^+^ metabolism enzyme, nicotinamide phosphoribosyltransferase (NAMPT, Figure 2E and Supplementary Figure S3) that resulted in a reduction of the cellular NAD^+^ concentration (Figure 2F). This consequently led to activation of *arf* transcription (Figure 2G) confirming that PAR synthesis by PARP1 leading to NAD^+^ depletion is required for the induction of *arf* transcription.

**Figure 3.**
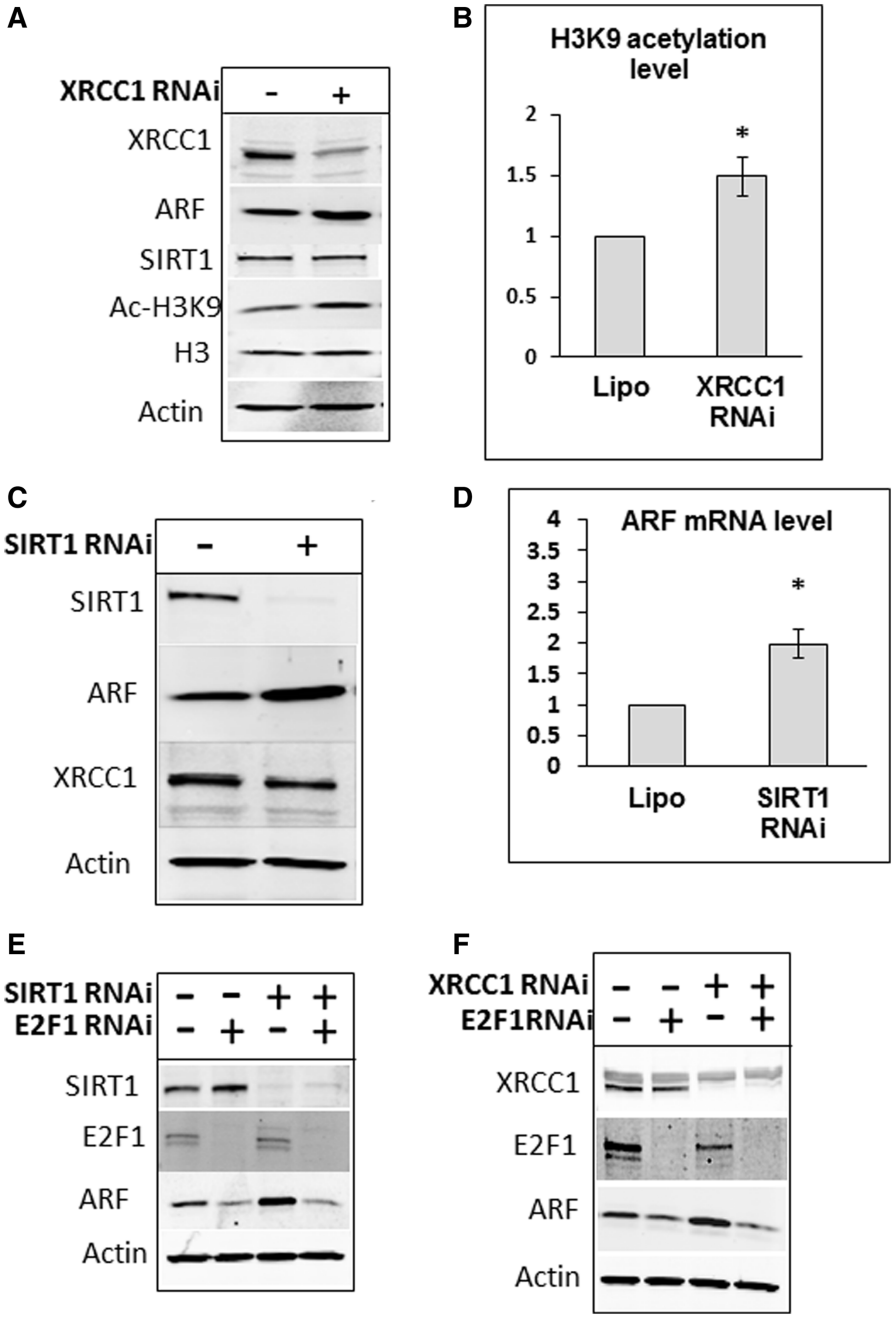
SIRT1 and E2F1 regulate *arf* transcription in response to unrepaired SBs. (**A**) XRCC1 knockdown reduces SIRT1 activity. HeLa cells were treated with Lipofectamine transfection reagent (10 µl) in the absence and presence of XRCC1 siRNA (200 pmol) for 72 h. Whole-cell extracts were prepared and analysed by SDS–PAGE and immunoblotting with the indicated antibodies. (**B**) Statistical analysis of the changes in histone H3 lysine 9 acetylation following an XRCC1 knockdown. Statistical data are presented as a mean ± SD of three independent biological experiments and *P*-values were calculated by Student’s *t*-test (*P < 0.05). (**C** and **D**) SIRT1 knockdown induces ARF protein levels and *arf* gene transcription. HeLa cells were treated with Lipofectamine transfection reagent in the absence and presence of SIRT1 siRNA (200 pmol) for 72 h. Whole-cell extracts were prepared and analysed by SDS–PAGE and immunoblotting with the indicated antibodies (C). mRNA was prepared and expression of *arf* gene was analysed by quantitative real-time PCR (D). (**E** and **F**) E2F1 is required for ARF accumulation in response to persistent SBs. HeLa cells were treated with Lipofectamine transfection reagent (10 µl) in the absence and presence of SIRT1, E2F1 (E) or XRCC1 (F) siRNA (200 pmol), or a combination of these, for 72 h. Whole-cell extracts were prepared and analysed by SDS–PAGE and immunoblotting with the indicated antibodies.

We consequently predicted that the NAD^+^-dependent deacetylase SIRT1 may play a role in the signal transmission pathway of persistent DNA damage, since the SIRT1-ARF axis was previously proposed to be activated in response to cellular stress, although the mechanism regulating this pathway was not clear (14,26). Since SIRT1 is NAD+-dependent enzyme, partial depletion of NAD+ after XRCC1 knockdown should result in decreased SIRT1 activity. To prove that SIRT1 activity was indeed decreased, we showed that induction of SBs by an XRCC1 knockdown results in increased acetylation of histone H3 at lysine 9 (Figure 3A and B), a well-known substrate for deacetylation by SIRT1 (36). These data suggest that consumption of NAD^+^ by PARP1 in response to persistent SBs reduces SIRT1 activity. To directly demonstrate that a reduction in SIRT1 activity results in ARF induction, we knocked down SIRT1 in HeLa cells and observed an up-regulation of *arf* transcription leading to elevated protein levels (Figure 3C and D). However, ARF protein stability remained the same following a SIRT1 knockdown (Supplementary Figure S4), suggesting that up-regulation of ARF protein levels by SIRT1 is mainly controlled at the transcriptional level.

It was previously demonstrated that SIRT1 deacetylates, and thus down regulates, the transcriptional activities of E2F1 (26). Therefore, we predicted that inactivation of SIRT1 observed in our experimental system should activate E2F1, which is known to be a transcription factor required for ARF expression (23–26). To confirm these findings, we showed that the induction of ARF protein following a SIRT1 siRNA knockdown is prevented by a simultaneous knockdown of E2F1 (Figure 3E). To prove the previously unknown link between SIRT1-E2F1 controlled ARF induction and persistent DNA SBs, we simultaneously knocked down XRCC1 and E2F1. We found that under these conditions, this prevents the elevation of ARF protein expression achieved with an XRCC1 knockdown alone (Figure 3F). Finally, to demonstrate E2F1-dependent activation of the *arf* promoter by SBs, we co-transfected cells with plasmids encoding E2F1 transcription factor and *arf* promoter-luciferase reporter plasmid. We observed an activation of E2F1-dependent *arf* transcription following an XRCC1 knockdown in comparison to control cells (Supplementary Figure S4). We thus concluded that SIRT1 and E2F1 are both involved in controlling *arf* gene transcription in response to persistent SBs, and that E2F1 is the final player in the DNA damage signal transmission pathway that is initiated by PARP1 protein.

### SB signalling pathway in normal human cells

**Figure 4.**
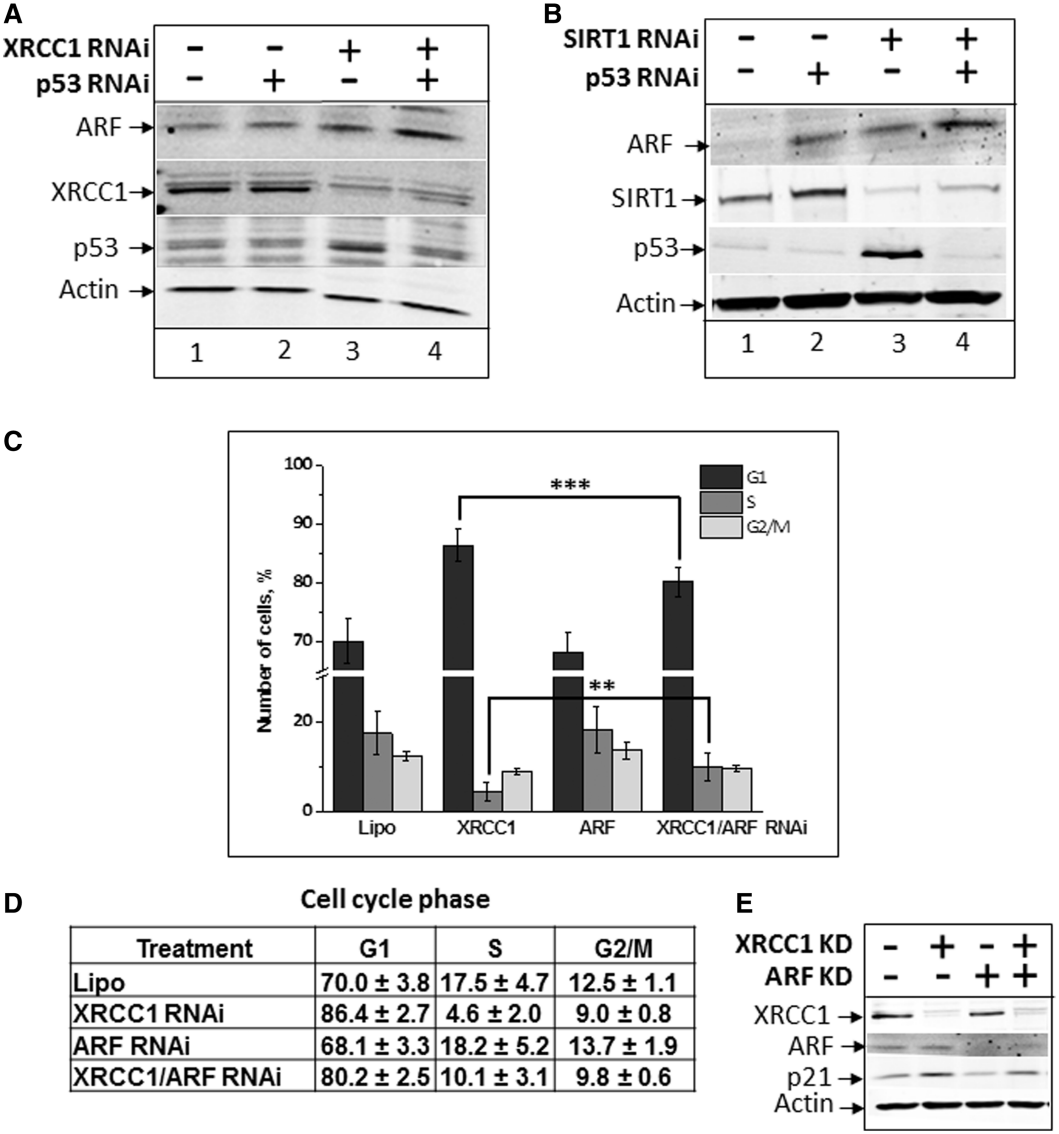
DNA SB signalling in normal cells. (**A** and **B**) TIG-1 cells (normal human foetal lung fibroblasts) were treated with Lipofectamine transfection reagent in the absence and presence of different combinations of XRCC1, p53 and SIRT1 siRNA (200 pmol) for 72 h. Whole-cell extracts were prepared and analysed by SDS–PAGE and immunoblotting with the indicated antibodies. **(C–E)** TIG-1 cells were treated with Lipofectamine transfection reagent in the absence (Lipo) or the presence of XRCC1, ARF or both siRNA (200 pmol each) for 72 h. (C–D) Cells were collected by trypsinization and subjected to FACS analysis. (E) Part of the cells from the same experiment were used for preparation of whole-cell extracts and western blot analysis.

We predicted that the DNA damage signal transmission pathway that we uncovered in HeLa cells should also operate in normal cells. We also predicted that this should occur on a much smaller scale since in normal cells the amplitude of ARF expression level is also controlled by a p53 negative feedback loop that inhibits *arf* transcription (37). Consequently, in primary human fibroblasts we observed that the induction of ARF protein following an siRNA knockdown of XRCC1 was quite moderate (Figure 4A, lane 3). However, it was markedly enhanced following a simultaneous knockdown of XRCC1 and p53 (Figure 4A, lane 4). In these cells, ARF was also slightly up-regulated in response to a SIRT1 knockdown (Figure 4B, lane 3), although again this was more pronounced in combination with a knockdown for p53 (Figure 4B, lane 4), indicating an important role for SIRT1 in down-regulating ARF protein expression in normal cells. As expected, both an XRCC1 knockdown (Figure 4A, lane 3), as well as a SIRT1 knockdown (Figure 4B, lane 3), induce p53 accumulation that limits the accumulation of ARF protein levels. Additional analysis of the expression of all genes involved SBs signalling is shown in Supplementary Figure S5.

Since it is widely accepted that as a part of the DNA damage response, cells would initiate a cell-cycle arrest to slow down cell proliferation therefore allowing time for repair of DNA damage, our data suggest that PARP1 deficient cells should be incapable of performing this step. Therefore, these cells will replicate damaged DNA without causing a cell-cycle delay. Indeed, knockdown of PARP1 resulted in a reduction of the amount of S-phase cells, suggesting that PARP1-deficient cells do not sense any unrepaired endogenous lesions and faster proceed through S-phase (Supplementary Figure S5). These data are supported by previous findings that PARP1 is required for replication fork slowing, or even reversion, in response to DNA damage (38,39). More importantly, cell-cycle delay in G1 phase following an XRCC1 knockdown was partially rescued by a simultaneous ARF knockdown (Figure 4C and D). An analysis of expression of the p21 protein that regulates cell-cycle progression further confirms the role of ARF in delaying cell cycle in response to accumulation of unrepaired SBs. We observed that while an XRCC1 knockdown induced the up-regulation of p21 expression, a simultaneous knockdown of ARF and XRCC1 reduced p21 expression by 30% (Figure 4E). This result is in a full agreement with the cell-cycle analysis data shown in Figure 4C. This suggests an important role for ARF in the DNA damage signalling pathway that we have discovered, in temporal coordination of DNA damage repair and replication.

**Figure 5.**
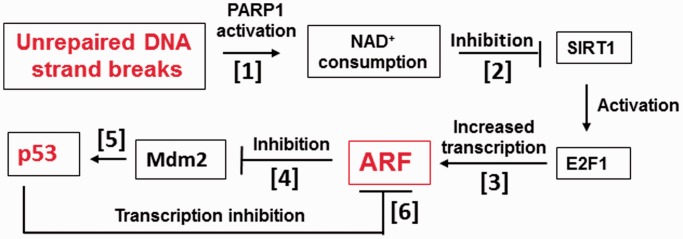
Proposed model for regulation of *arf* gene transcription in response to unrepaired SBs. Consumption of NAD^+^ by PARP1 (1) at the sites of SBs results in inhibition of the NAD^+^-dependent deacetylase SIRT1 (2), leading to activation of the E2F1 transcription factor and increase in ARF expression (3). This results in inhibition of Mdm2 (4) and accumulation of p53 protein (5). Importantly, amplitude of the signal is controlled by the p53 protein feedback loop (6), most probably regulating the decision between cellular life or death (replication or apoptosis).

Taken together, our data provide a mechanism for the detection and quantitative reporting of unrepaired DNA damage to cellular stress response proteins (Figure 5). This is achieved by consumption of NAD^+^ by PARP1 at the sites of SBs, which results in inhibition of the NAD^+^-dependent deacetylase SIRT1, leading to activation of the E2F1 transcription factor and consequent increase in ARF and p53 protein expression. Importantly, amplitude of the signal is controlled by the p53 protein feedback loop, most probably regulating the decision between cellular life or death (replication or apoptosis).

## DISCUSSION

ARF is the major protein that regulates p53 stability by inhibiting the E3 ubiquitin ligase Mdm2 (4,13,40). It has been previously established that ARF is regulated both at transcriptional and post-translational levels. At the post-translational level, ARF stability is regulated by the E3 ubiquitin ligase ULF, although no links between the cellular response to DNA damage, and the mechanisms regulating ULF activity, have been reported suggesting that ULF is mainly controlling cellular steady state levels of ARF (29). At the transcriptional level, expression of the *arf* gene is regulated by the transcription factors E2F1 and p53, which have been shown to activate and down-regulate *arf* transcription, respectively (37,40). However, the stimuli regulating *arf* gene transcription and the mechanism(s) involved remained unclear. Since published data on ARF protein induction by DNA damaging agents were inconsistent, oncogenes, such as c-Myc, were the only recognized ARF inducers (11), although the observation that c-Myc may induce DNA damage (41,42) was not widely accepted. This led to a paradoxical situation since it is well established that p53, the major target of ARF, is induced in response to DNA damage and at the same time ARF was thought not to be responsive to DNA damage. We now demonstrate that ARF is induced in response to unrepaired SBs and we have identified the mechanism transmitting the DNA damage signal to the *arf* gene. In our opinion, several factors have prevented the discovery of the mechanism of ARF induction by DNA damage. First, as we demonstrated in this study, DNA damage induction through chemical modification does not induce NAD^+^ consumption and consequently would also not activate *arf* transcription. Furthermore, we demonstrated that only unrepaired SBs can induce *arf* transcription, since the pathway is activated by NAD^+^ depletion through PARP1 that synthesizes PAR polymers at the sites of SBs. Second, to induce substantial NAD^+^ depletion required for detectable ARF induction, the amount of SBs should be above the repair capacity of the cells. Third, normal cells control ARF induction through the p53 feedback loop that significantly limits the amplitude of ARF protein levels (Figure 4A and B).

The existence of the p53 feedback loop suggests that the generation of SBs and their repair is a dynamic process that will result in attenuated pulses of ARF induction, following by attenuated pulses of p53 protein levels. Taking into account the fact that genomic DNA damage, including formation of SBs, is an ongoing process due to the chemical instability of the DNA molecule and endogenous mutagens (34), this may lead to multiple repeated pulses of ARF and p53 protein levels before cells would be able to proceed with replication. Indeed, analysis of the dynamics of p53 protein levels has revealed attenuated pulses of p53 induction in cycling cells (43). Most probably, such a delicate regulation of cellular p53 protein levels is required as a buffer that prevents cells undergoing apoptosis each time DNA damage is detected.

Since the SB signalling pathway uncovered in this study is regulated by the cellular concentration of NAD^+^, our findings have revealed an important link between DNA damage, cellular metabolism and cancer. We speculate that the recognition of the cellular levels of NAD^+^, as a major indicator of the cellular DNA damage landscape and regulator of the DNA damage response, provides a mechanistic link explaining the role of dietary restrictions in genome stability and cancer. Our data support the idea that modulation of NAD^+^ levels by specific caloric diet will in turn modulate sensitivity of the DNA damage signal transduction pathway, since it is regulated by the cellular concentration of NAD^+^. An excess of NAD^+^ (high calorie diet) will decrease the sensitivity of the pathway and as a consequence will allow replication of damaged DNA and may potentially be a cancer risk factor. Second, the role of PARP1 and SIRT1 in the protein cascade that transmits the DNA damage signal suggests that artificial modulation of these protein levels and activities may also be an important risk factor. According to our model, both reduction of PARP1 activity by inhibitors or artificial stimulation of SIRT1 activity will result in reduced sensitivity of the DNA damage reporting system and potentially result in the accumulation of DNA damage, and promote tumorigenesis. This idea is supported by the finding that a deficiency in PARP1 accelerates spontaneous carcinogenesis in mice (44).

In summary, our data provide a molecular mechanism for DNA damage signal transmission that links together DNA quality control, metabolism and cancer.

## SUPPLEMENTARY DATA

Supplementary Data are available at NAR Online, including [45–52].

## FUNDING

The Medical Research Council and Cancer Research UK. Funding for open access charge: Medical Research Council.

*Conflict of interest statement*. None declared.

## Supplementary Material

Supplementary Data
