# Correction to “ARF induction in response to DNA strand breaks is regulated by PARP1”

**DOI:** 10.1093/nar/gkag013

**Published:** 2026-01-14

This is a correction to: Giulia Orlando, Svetlana V. Khoronenkova, Irina I. Dianova, Jason L. Parsons, Grigory L. Dianov, ARF induction in response to DNA strand breaks is regulated by PARP1, *Nucleic Acids Research*, Volume 42, Issue 4, 1 February 2014, Pages 2320–2329, https://doi.org/10.1093/nar/gkt1185

In November 2025, the Editors were alerted to a duplication in the PAR and ARF immuno-blots in Figures 2A and 1C. The authors have acknowledged this oversight and have provided a replacement figure for Figure 2A (see below). The original data are provided as a supplementary file. The authors have highlighted that PAR and ARF levels, following an XRCC1 siRNA, are shown to be increased in two additional independent experiments within the paper, including as part of a time course experiment, demonstrated in Figure 1G and Figure 2C.

This correction does not affect the results, discussion and conclusions presented in the article. The figure has been corrected only in this correction notice to preserve the published version of record.


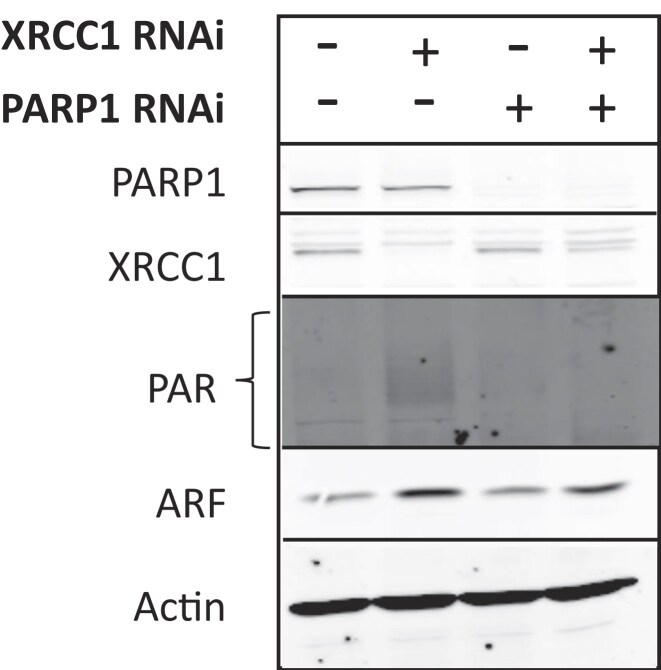


**Updated Figure 2A**

## Supplementary Material

gkag013_Supplemental_File

