# Supplementary material for: ARF induction in response to DNA strand breaks is regulated by PARP1
Source: Nucleic Acids Res. 2013 Nov 29;42(4):2320–9. doi: 10.1093/nar/gkt1185 (PMC3936746; doi:10.1093/nar/gkt1185)
Supplement: Supplementary Data [file supp_42_4_2320__index.html]

ARF induction in response to DNA strand breaks is regulated by PARP1 — ARF induction in response to DNA strand breaks is regulated by PARP1 — Supplementary Data 

# ARF induction in response to DNA strand breaks is regulated by PARP1

## Supplementary Data

files

**Files in this Data Supplement:**

- Supplementary Data - pdf file
