# Supplementary material for: ARF induction in response to DNA strand breaks is regulated by PARP1
Source: Nucleic Acids Res. 2013 Nov 29;42(4):2320–9. doi: 10.1093/nar/gkt1185 (PMC3936746; doi:10.1093/nar/gkt1185)
Supplement: Supplementary Data [file supp_gkt1185_nar-01484-d-2013-File007.pdf]

Supplemental information

ARF induction in response to DNA strand breaks is regulated by PARP1

Giulia Orlando, Svetlana V Khoronenkova, Irina I Dianova, Jason L Parsons[1] and Grigory L Dianov*

Gray Institute for Radiation Oncology and Biology, Department of Oncology, University of Oxford, Roosevelt Drive, Oxford OX3 7DQ, UK

[1]Current address: Department of Molecular and Clinical Cancer Medicine, Cancer Research Centre, University of Liverpool, 200 London Road, Liverpool L3 9TA, UK

*To whom correspondence should be addressed: Gray Institute for Radiation Oncology and Biology, University of Oxford, Old Road Campus Research Building, Roosevelt Drive, Oxford OX3 7DQ, UK

Tel: (44) 1865617325; email: grigory.dianov@oncology.ox.ac.uk

SUPPLEMENTAL FIGURES

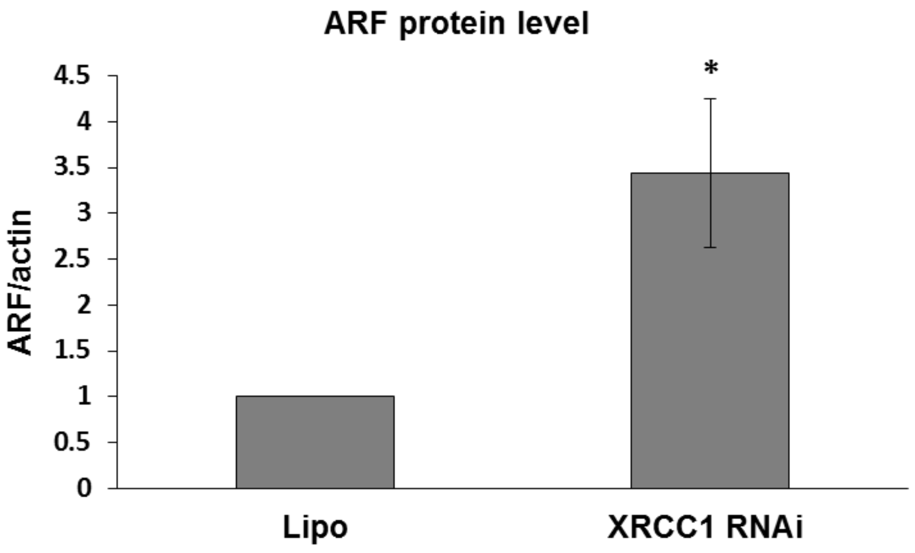

Figure S1, related to Fig. 1. Quantification of ARF induction after XRCC1 knockdown. Western blot bands of ARF protein from four independent experiments have been quantified using LICOR software and actin has been used as a loading control. Statistical data are presented as a mean ± S.D. of 4 independent biological experiments and p-values were calculated by the Student's t test (*p<0.05).

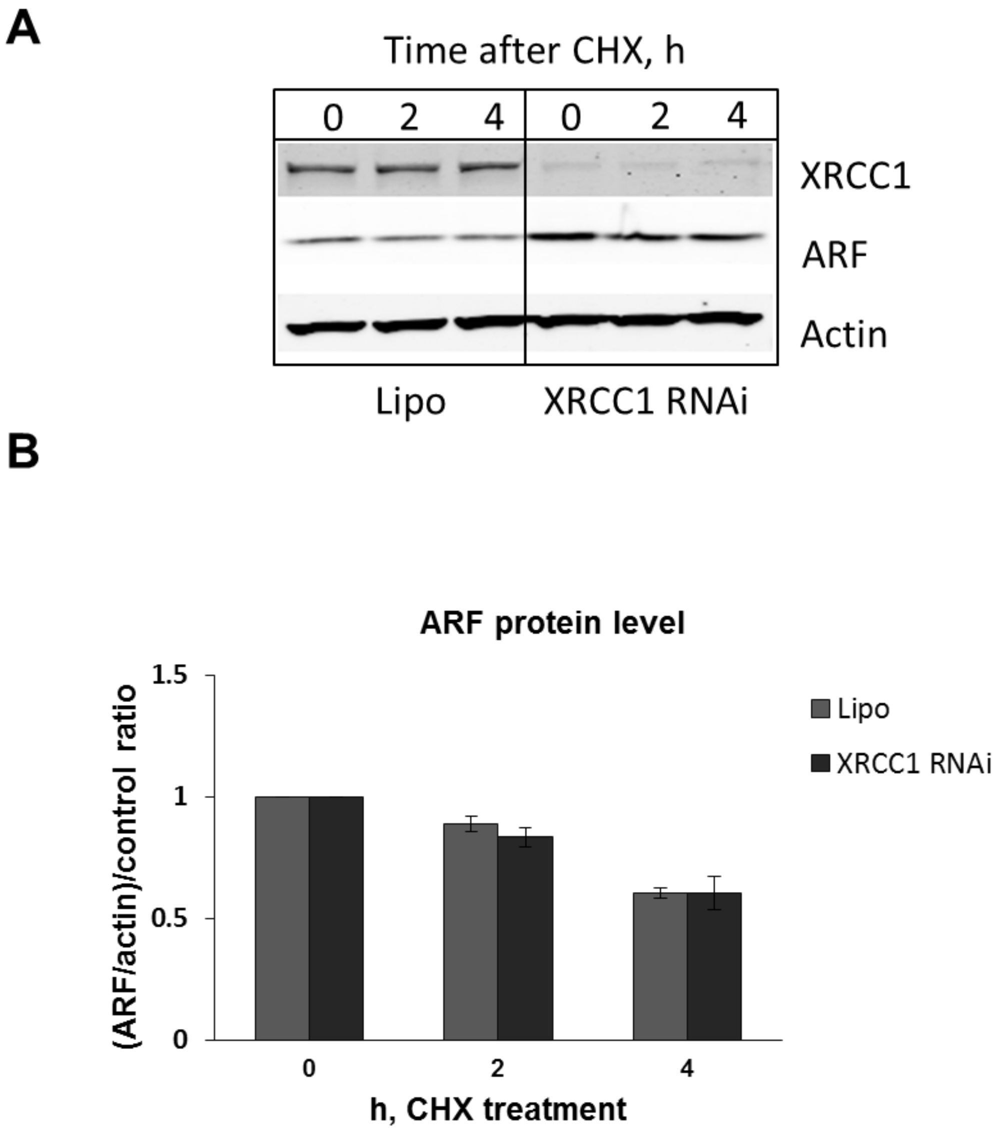

Figure S2, related to Fig. 1. ARF accumulation following XRCC1 knockdown is not due to increased protein stability.

A, HeLa cells were treated with Lipofectamine transfection reagent in the presence of XRCC1 (0.2 pmol) siRNA for 72 h. Cells were then treated with 50 µg/ml cycloheximide for 0, 2 or 4 h and pelleted by centrifugation. Whole cell extracts were prepared and analysed by Western blotting. **B,** The data are presented graphically as a mean ± S.D. of 3 independent biological experiments.

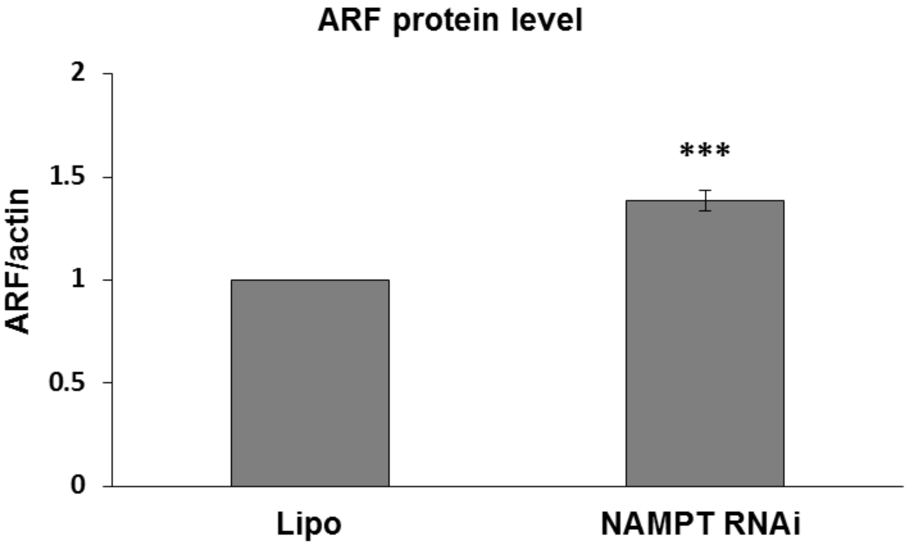

Figure S3, related to Fig. 2. Quantification of ARF induction after NAMPT knockdown. HeLa cells were treated with Lipofectamine transfection reagent in the absence and presence of NAMPT siRNA (200 pmol) for 72 h. Whole cell extracts were prepared and analysed by SDS-PAGE. Western blot bands of ARF protein from four independent experiments have been quantified using LICOR software and actin has been used as loading control. Statistical data are presented as a mean ± S.D. of 3 independent biological experiments and p-values were calculated by Student's t test (***p<0.001).

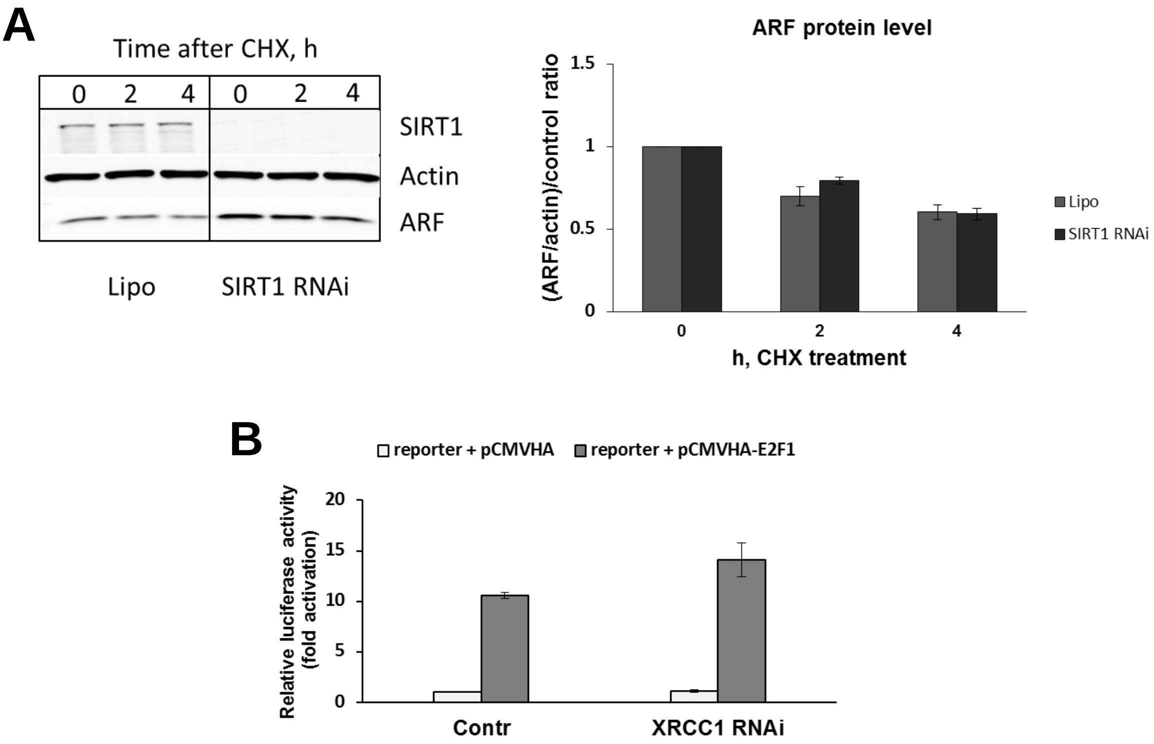

Figure S4, related to Fig. 3. (A) ARF accumulation after SIRT1 knockdown is not due to increased protein stability. (B) Activation of *ARF* promoter by E2F1 in response to XRCC1 knockdown. (**A**) HeLa cells were treated with Lipofectamine transfection reagent in the presence of SIRT1 siRNA (0.2 pmol) for 72 h. Cells were then treated with 50 µg/ml cycloheximide for 0, 2 or 4 h and pelleted by centrifugation. Whole cell extracts were prepared and analysed by Western blotting. The data are presented graphically as a mean ± S.D. of 3 independent biological experiments. (**B**) HeLa cells were treated with Lipofectamine transfection reagent in the absence (Contr) or presence of XRCC1 siRNA (0.2 pmol, XRCC1 RNAi) for 48 h and further transfected with p14ARF promoter luciferase reporter plasmid (200 ng), pCMVHA-E2F1 or control empty vector (200 ng), and pRL-TK Renilla luciferase reporter plasmid (10 ng) as an internal control using Lipofectamine transfection reagent for 24 h. Cells were then lysed and subjected to analysis using a luciferase assay. Statistical data are presented as a mean ± S.D. of 3 independent biological experiments and the p-value was calculated by Student's t-test (*$p<0.05$).

A

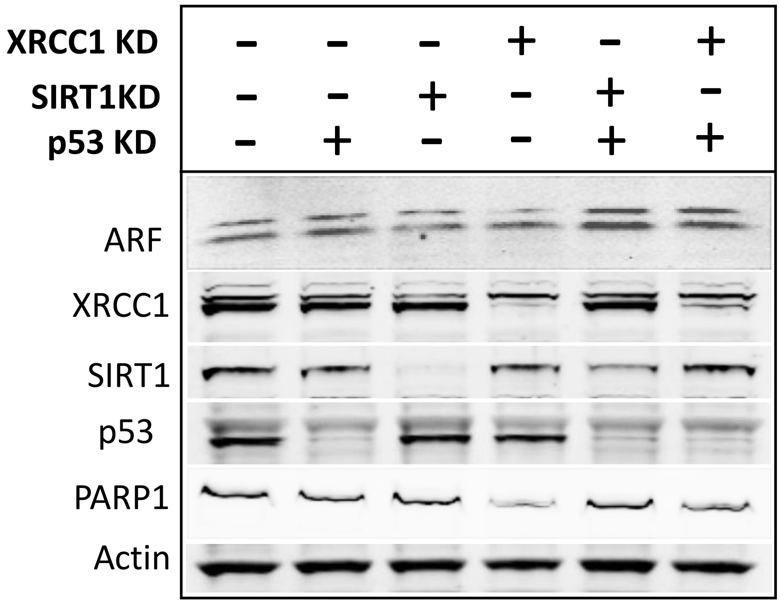

B

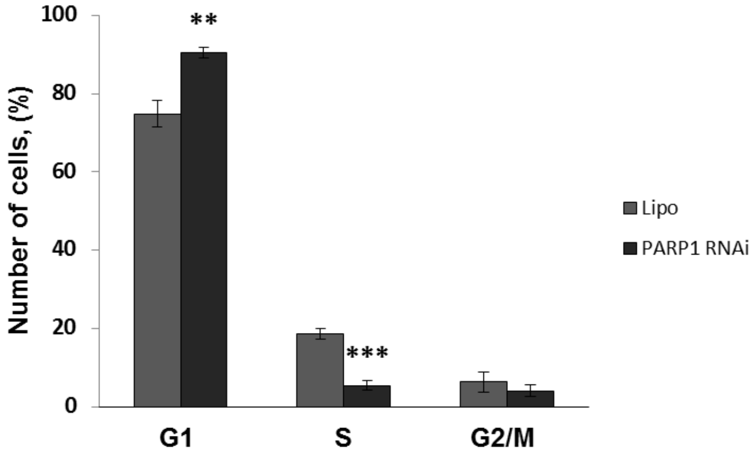

Figure S5, related to Fig.4. (A) Protein expression profile after knockdown of genes involved in SB signalling in AG06173 cells. AG06173 cells were treated with Lipofectamine transfection reagent in the absence and presence of different combinations of XRCC1, p53 and SIRT1 siRNA (200 pmol) for 72 h. Whole cell extracts were prepared and analysed by SDS-PAGE and immunoblotting with the indicated antibodies. **(B) Reduction in PARP1 levels after XRCC1 knockdown reflects it poly(ADP-ribosyl)ation. PARP1 is required for DNA damage-dependent cell cycle delay.** AG06173 cells were treated with

Lipofectamine transfection reagent in the absence (Lipo) or the presence of PARP1 siRNA (200 pmol) for 72 h. Cells were collected by trypsinisation and subjected to FACS analysis. Statistical data are presented as a mean ± S.D. of 3 independent biological experiments and the p-values were calculated by Student's t-test (**$p<0.01$, ***$p<0.001$).

Table 1. Antibodies employed in this work

Protein	Provider	Product code
XRCC1	Abcam	ab144
XRCC1	custom generated to full length protein	
PARP1	custom generated to full length protein	
SIRT1	Santa Cruz	sc-15404
p53	Santa Cruz	sc-126
E2F1	Santa Cruz	C-20/sc-193
E2F1	Santa Cruz	sc-251
Actin	Abcam	ab6276
ARF	Axxora	BET-A300-340A
PAR	Trevigen	4335-AMC-050
AcH3K9	Cell Signalling	9649
H3	Cell Signalling	9715
NAMPT	Bethyl	A300-779A
APE1	custom generated to full length protein	

Table 2. siRNA sequences used for knock down experiments

Gene	Sequence	References
XRCC1	5'-AGGGAAGAGGAAGUUGGAU-3'	(Brem and Hall 2005)
SIRT1	5'-GAUGAAGUUGACCUCCUCA-3'	(Lee et al. 2011)
p53	5'-AAGACUCCAGUGGUAAUCUAC-3'	(Zhu et al. 2004)
E2F1	5'-CGCUAUGAGACCUCACUGA-3'	(Goto et al. 2006)
PARP1	5'-AAGAUAGAGCGUGAAGGCGAA-3'	(Kameoka et al. 2004)
NAMPT	5'-GAGUGUUACUGGCUUACAA-3'	(Zhang et al. 2009)
APE1	5'-AAUGACAAAGAGGCAGCAGG-3'	(Fung and Demple 2005)
APE1	5'-AACCUGCCACACUCAAGAUC-3'	(Fung and Demple 2005)
ARF	5'-GAACAUGGUGCGCAGGUUCTT-3'	(Eymin et al. 2006)

SUPPLEMENTAL REFERENCES

Brem R, Hall J. 2005. XRCC1 is required for DNA single-strand break repair in human cells. *Nucleic Acids Res* **33**: 2512-2520.

Eymin B, Claverie P, Salon C, Leduc C, Col E, Brambilla E, Khochbin S, Gazzeri S. 2006. p14ARF activates a Tip60-dependent and p53-independent ATM/ATR/CHK pathway in response to genotoxic stress. *Mol Cell Biol* **26**: 4339-4350.

Fung H, Demple B. 2005. A vital role for ape1/ref1 protein in repairing spontaneous DNA damage in human cells. *Mol Cell* **17**: 463-470.

Goto Y, Hayashi R, Kang D, Yoshida K. 2006. Acute loss of transcription factor E2F1 induces mitochondrial biogenesis in HeLa cells. *J Cell Physiol* **209**: 923-934.

Kameoka M, Nukuzuma S, Itaya A, Tanaka Y, Ota K, Ikuta K, Yoshihara K. 2004. RNA interference directed against Poly(ADP-Ribose) polymerase 1 efficiently suppresses human immunodeficiency virus type 1 replication in human cells. *J Virol* **78**: 8931-8934.

Lee YM, Shin SI, Shin KS, Lee YR, Park BH, Kim EC. 2011. The role of sirtuin 1 in osteoblastic differentiation in human periodontal ligament cells. *J Periodontal Res* **46**: 712-721.

Zhang T, Berrocal JG, Frizzell KM, Gamble MJ, DuMond ME, Krishnakumar R, Yang T, Sauve AA, Kraus WL. 2009. Enzymes in the NAD+ salvage pathway regulate SIRT1 activity at target gene promoters. *J Biol Chem* **284**: 20408-20417.

Zhu W, Chen Y, Dutta A. 2004. Rereplication by depletion of geminin is seen regardless of p53 status and activates a G2/M checkpoint. *Mol Cell Biol* **24**: 7140-7150
