# Supplementary figures and images for: Correction to “ARF induction in response to DNA strand breaks is regulated by PARP1”
Source: Nucleic Acids Res. 2026 Jan 14;54(2):gkag013. doi: 10.1093/nar/gkag013 (PMC12802906; doi:10.1093/nar/gkag013)

## Slide 1
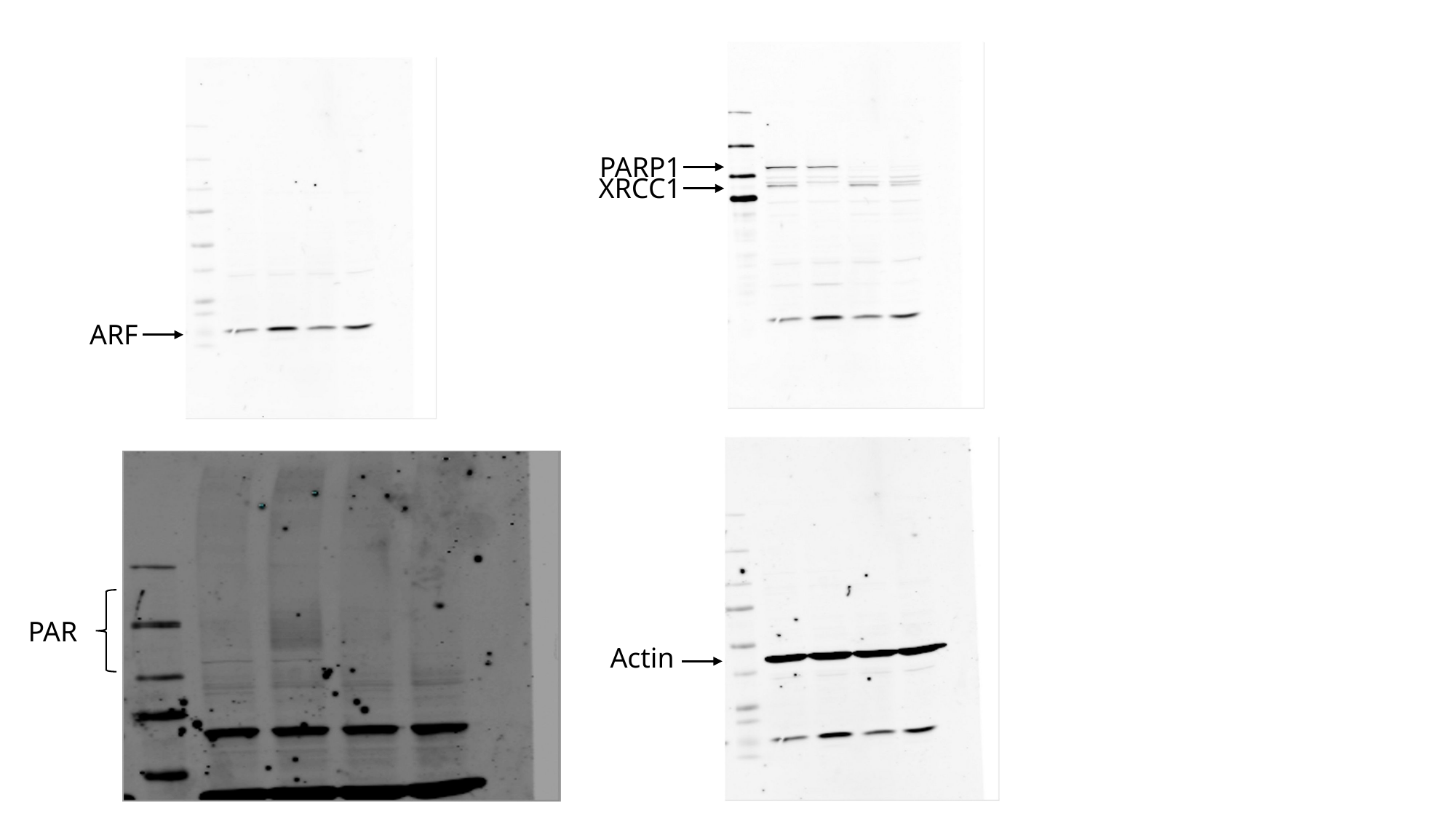

PARP1
XRCC1
ARF
PAR
Actin

Supplement: gkag013_Supplemental_File [file gkag013_supplemental_file.pptx]
